# An open-source repository-based tool for quality control of imaging protocol compliance: demonstration in a multicentre MRI study

**DOI:** 10.1093/bjr/tqaf089

**Published:** 2025-06-10

**Authors:** Sam Keaveney, Damien J McHugh, Mihaela Rata, Alina Dragan, Jessica M Winfield, Simon J Doran, Matthew D Blackledge, Erica Scurr, Dow-Mu Koh, Michael Berks, Andrew B Gill, Jonathan R Birchall, James P B O’Connor, Alexander King, Winston J Rennie, Suchi Gaba, Priya Suresh, Paul Malcolm, Amy Davis, Anjumara Nilak, Aarti Shah, Sanjay Gandhi, Mauro Albrizio, Guy Pratt, Gordon Cook, Andrew Hall, Sadie Roberts, Matthew Jenner, Sarah Brown, Martin Kaiser, Penny L Hubbard Cristinacce, Christina Messiou

**Affiliations:** MRI Unit, The Royal Marsden NHS Foundation Trust, London, SM2 5PT, United Kingdom; Division of Radiotherapy and Imaging, The Institute of Cancer Research, London, SW3 6JB, United Kingdom; Christie Medical Physics and Engineering, The Christie NHS Foundation Trust, Manchester, M20 4BX, United Kingdom; Quantitative Biomedical Imaging, Division of Cancer Sciences, University of Manchester, Manchester, M14 4PX, United Kingdom; MRI Unit, The Royal Marsden NHS Foundation Trust, London, SM2 5PT, United Kingdom; Division of Radiotherapy and Imaging, The Institute of Cancer Research, London, SW3 6JB, United Kingdom; MRI Unit, The Royal Marsden NHS Foundation Trust, London, SM2 5PT, United Kingdom; MRI Unit, The Royal Marsden NHS Foundation Trust, London, SM2 5PT, United Kingdom; Division of Radiotherapy and Imaging, The Institute of Cancer Research, London, SW3 6JB, United Kingdom; MRI Unit, The Royal Marsden NHS Foundation Trust, London, SM2 5PT, United Kingdom; Division of Radiotherapy and Imaging, The Institute of Cancer Research, London, SW3 6JB, United Kingdom; MRI Unit, The Royal Marsden NHS Foundation Trust, London, SM2 5PT, United Kingdom; Division of Radiotherapy and Imaging, The Institute of Cancer Research, London, SW3 6JB, United Kingdom; MRI Unit, The Royal Marsden NHS Foundation Trust, London, SM2 5PT, United Kingdom; MRI Unit, The Royal Marsden NHS Foundation Trust, London, SM2 5PT, United Kingdom; Division of Radiotherapy and Imaging, The Institute of Cancer Research, London, SW3 6JB, United Kingdom; Quantitative Biomedical Imaging, Division of Cancer Sciences, University of Manchester, Manchester, M14 4PX, United Kingdom; Department of Radiology, University of Cambridge, Cambridge, CB2 0QQ, United Kingdom; Department of Radiology, Royal Papworth Hospital NHS Foundation Trust, Cambridge, CB2 0AY, United Kingdom; Department of Radiology, University of Cambridge, Cambridge, CB2 0QQ, United Kingdom; Division of Radiotherapy and Imaging, The Institute of Cancer Research, London, SW3 6JB, United Kingdom; Quantitative Biomedical Imaging, Division of Cancer Sciences, University of Manchester, Manchester, M14 4PX, United Kingdom; Department of Radiology, The Christie NHS Foundation Trust, Manchester, M20 4BX, United Kingdom; Department of Haematology, University Hospital Southampton NHS Foundation Trust, Southampton, SO16 6YD, United Kingdom; Department of Radiology, University Hospitals of Leicester NHS Trust, Leicester, LE1 5WW, United Kingdom; Department of Radiology, University Hospitals of North Midlands NHS Trust, ST4 6QG, United Kingdom; Department of Radiology, University Hospitals Plymouth NHS Trust, Plymouth, PL6 8DH, United Kingdom; Department of Radiology, Norfolk & Norwich University Hospitals NHS Foundation Trust, Norwich, NR4 7UY, United Kingdom; Department of Radiology, Epsom & St Helier University Hospitals NHS Trust, Epsom, KT18 7EG, United Kingdom; Department of Radiology, Worcestershire Acute Hospitals NHS Trust, Worcester, WR5 1DD, United Kingdom; Department of Radiology, Hampshire Hospitals NHS Foundation Trust, Basingstoke, RG24 9NA, United Kingdom; Department of Radiology, North Bristol NHS Trust, Bristol, BS10 5NB, United Kingdom; Department of Radiology, Nottingham University Hospitals NHS Trust, Nottingham, NG5 1PB, United Kingdom; Department of Haematology, University Hospitals Birmingham NHS Foundation Trust, Birmingham, B15 2GW, United Kingdom; Clinical Trials Research Unit, Leeds Institute of Clinical Trials Research, University of Leeds, Leeds, LS2 9NL, United Kingdom; Leeds Cancer Centre, Leeds Teaching Hospitals NHS Trust, Leeds, LS9 7TF, United Kingdom; Clinical Trials Research Unit, Leeds Institute of Clinical Trials Research, University of Leeds, Leeds, LS2 9NL, United Kingdom; Clinical Trials Research Unit, Leeds Institute of Clinical Trials Research, University of Leeds, Leeds, LS2 9NL, United Kingdom; Department of Haematology, University Hospital Southampton NHS Foundation Trust, Southampton, SO16 6YD, United Kingdom; Clinical Trials Research Unit, Leeds Institute of Clinical Trials Research, University of Leeds, Leeds, LS2 9NL, United Kingdom; Division of Genetics and Epidemiology, The Institute of Cancer Research, London, SW3 6JB, United Kingdom; Department of Haematology, The Royal Marsden NHS Foundation Trust, London, SM2 5PT, United Kingdom; Quantitative Biomedical Imaging, Division of Cancer Sciences, University of Manchester, Manchester, M14 4PX, United Kingdom; MRI Unit, The Royal Marsden NHS Foundation Trust, London, SM2 5PT, United Kingdom; Division of Radiotherapy and Imaging, The Institute of Cancer Research, London, SW3 6JB, United Kingdom

**Keywords:** magnetic resonance imaging, quality control, software, multiple myeloma, whole-body imaging, clinical translation

## Abstract

**Objectives:**

Clinical translation of advanced MRI techniques can be hindered by the challenges of performing standardized multicentre imaging trials. This work aims to develop and demonstrate an automated tool for monitoring imaging protocol deviations, enabling corrective action to be taken.

**Methods:**

A Python-based tool, integrated into the imaging repository XNAT, was developed to compare DICOM series with an agreed imaging protocol, highlighting missing series and parameter deviations. This was demonstrated through retrospective analysis of a prospectively acquired dataset from a ten-site whole-body (WB) MRI study of patients with multiple myeloma. The acquired data were compared to the relevant radiological guidelines and to the site-specific imaging protocols agreed for the study.

**Results:**

The rate of technical software failure was 0% across 174 examinations from 10 sites. The clinical guidelines were followed in 87.9% of examinations and compliance with the site-specific imaging protocol was greater than 75.0% for all parameters. Common deviations included number of averages for diffusion-weighted imaging (DWI) and repetition time for DWI and Dixon: 85.2%, 81.7%, and 75.1%, respectively. There was a statistically significant correlation between protocol compliance and overall exam radiological image quality.

**Conclusions:**

Repository-integrated software is presented for automated monitoring of imaging protocol compliance to support standardization in multicentre studies and clinical translation.

**Advances in knowledge:**

This study presents a novel open-source repository-integrated software tool for automatically monitoring compliance with the expected imaging protocol. Standardized acquisition protocols are crucial in multicentre imaging studies and this tool has the potential to enhance research outcomes and support clinical translation.

## Introduction

Multicentre studies are a crucial step in the translation of quantitative MR imaging biomarkers from academic research into clinical practice.^1–3^ To ensure that study data are evaluable and analysis outcomes are robust, it is essential that acquisitions are performed according to the study imaging protocol.

Multicentre studies should include a site qualification process to establish an imaging protocol at each site capable of acquiring images of sufficient quality. Critically, all images should be acquired in accordance with this protocol.^3^^,^^4^ In clinical research, there may be valid reasons to change some acquisition parameters (eg, to reduce acquisition time for a patient in discomfort or to adapt the imaging protocol to lower technical specifications of a particular scanner). However, certain parameters must be kept consistent for the results of quantitative analysis to be comparable between patients and across multiple scanning sessions for the same patient.

Many aspects of imaging protocol compliance can be monitored using the DICOM attributes of the imaging data^5^; however, this process is time-consuming and error-prone if performed manually. An automated method is required to conduct these checks in a robust standardized manner,^6^ particularly for large datasets. Although software solutions exist for this, they are generally not open-source or easily configurable by the user. XNAT is an open-source informatics platform^7^ that is widely used to support multicentre studies^8–10^ and supports the integration of user-defined containerized software for data quality control (QC), image processing, and analysis. This work presents a protocol compliance tool that has been implemented within XNAT and can be used to: (a) conduct a large-scale retrospective evaluation of imaging protocol compliance; and/or (b) perform automated checks on images as they arrive in the repository throughout the study, allowing sites to be given rapid feedback regarding imaging protocol compliance and enabling corrective action to be taken. Although it is presented here as part of a pipeline in XNAT, the software can also be used as a standalone tool or with other image repositories that can integrate containerized code.

We sought to demonstrate the value of an automated XNAT-integrated protocol compliance tool, hereafter referred to as Protocol Checker, in a multicentre whole-body (WB) MRI study. National and international guidelines recommend WB-MRI as first-line imaging for patients with a suspected diagnosis of myeloma,^11^^,^^12^ and the importance of standardized acquisition is reflected in the publication of the Myeloma Response Assessment and Diagnosis System (MY-RADS) guidelines for WB-MRI in myeloma.^13^ MY-RADS recommends key imaging parameters for WB diffusion-weighted imaging (DWI), T1-weighted (T1w) Dixon imaging and T1 and T2-weighted (T2w) sagittal spine imaging. The Myeloma UK nine OPTIMUM trial (MUKnine) is a multicentre study applying WB-MRI, acquired in accordance with the MY-RADS guidelines, to monitor treatment response in patients with multiple myeloma.^14^ Sites enrolled in the MUKnine study underwent a qualification process to establish a MY-RADS-compliant imaging protocol on their local scanner, with some protocol deviations agreed in advance for specific sites due to hardware and scan time limitations.^15^ The sites enrolled to MUKnine include a range of scanner hardware, software and prior experience of WB-MRI (including sites with no prior experience), reflecting the situation that is often found in clinical practice. The establishment of a standardized imaging protocol across a range of scanners was a key aim of MUKnine.

This work describes the design and use of Protocol Checker and demonstrates its application in a retrospective analysis of the prospectively acquired MUKnine dataset to perform an audit of compliance with the MY-RADS guidelines and with site-specific imaging protocols set up during site qualification. The software is demonstrated with WB-MRI data; however, it is designed to be versatile and can be applied to any imaging data in the DICOM format.

## Methods

Protocol Checker was developed in Python (v3.9.13, Python Software Foundation, Wilmington DE, USA) to perform automated imaging protocol compliance checks. The package performs 2 functions: during site qualification, it generates a master file that defines the expected imaging protocol using 1 scanning session as a template; and throughout the study, it performs compliance checks by comparing the newly acquired examinations to the created master file. The functions are performed by the commands *Generate master file* and *Scan protocol check*, respectively.

### Generate master file

This is run on an exemplar scanning session that is known to have been acquired using the desired imaging protocol to generate the master file, which specifies the expected series and values for pre-determined parameters. This function takes as an input a user-defined text file, known as the attribute file, containing a list of DICOM metadata tags that are considered of interest for the study. The function performs the following actions on the imaging data:

1. Sorts DICOM files into series according to the DICOM metadata tags Series Number (0020,0011), Acquisition Number (0020,0012), Slice Location (0020,1041), and Temporal Position Identifier (0020,0100).
2. For each series, extracts the DICOM metadata tag that identifies the type of imaging sequence. This tag varies by manufacturer, but possible fields include Sequence Name (0018,0024), Pulse Sequence Name (0018,9005), or Scanning Sequence (0018,0020). Further information to distinguish series is extracted from the DICOM metadata tag Series Description (0008,103E), although this can be modified at the time of scan so may be less reliable.
3. Extracts generic attributes about the acquisition from the DICOM metadata tags Modality (0008,0060), Magnetic Field Strength (0018,0087), Manufacturer (0008,0070), and Manufacturer Model Name (0008,1090).
4. For each series, extracts series-specific attributes, that is, the value of each of the parameters listed in the attribute file.
5. Writes this information out in JavaScript Object Notation (JSON)-format file with defined structure (the master file), which is saved as an output.

The user then manually checks the master file against the agreed imaging protocol to ensure the exemplar data were acquired correctly, making any necessary changes manually. It is possible to state expected parameter values exactly or to define an acceptable range by replacing the exact value with minimum and maximum values (eg, to specify that the TR should be between 6.0 s and 6.5 s, the following syntax would be used: “RepetitionTime”: {“high”: 6500, “low”: 6000}). The acceptable range should be determined prospectively at the beginning of the study, ideally with reference to quantitative analysis outcome measures or image quality.^3^ The master file is then used as an input for the command *Scan protocol check.*

### Scan protocol check

This uses the study- and site-specific master file to compare each subsequently acquired imaging session with the expected imaging protocol, performing the following actions:

1. Sorts DICOM files into series in the same way as the previous function.
2. Categorizes the series as follows:
  - For each series, extracts the DICOM tag that identifies the type of imaging sequence (eg, Sequence Name (0018,0024), although this will vary by manufacturer as described previously). This tag is used to match the series to an entry in the master file, identifying any missing or unexpected series.
  - The value of this tag may be relatively generic, for example, it may simply identify the sequence as a turbo spin echo. An imaging protocol may contain multiple series with a turbo spin echo base sequence, so further information is required to distinguish between series. In these cases, the Series Description (0008,103E) tag is used for additional discrimination. As this field is user-modifiable at the time of acquisition, only a partial match with the value in the master file is required.
3. Extracts the value of the generic attributes from the DICOM metadata and compares with those stated in the master file.
4. For each series, compares the value of each parameter listed in the master file to that in the DICOM metadata of the new data, identifying where parameter discrepancies have occurred.
5. Produces a text file summarizing the missing/unexpected series and any parameter deviations.

Protocol Checker can be used offline as a stand-alone application or integrated into an imaging repository such as XNAT. Protocol Checker was containerized using Docker (v20.10.5, Docker, Inc., Palo Alto CA, USA), and the XNAT container service plugin^16^ (v3.1.1) was used to pull the Docker image onto a central XNAT node (v1.8.9) which acted as the data repository for MUKnine. The Protocol Checker code repository and Docker image can be accessed at https://gitlab.com/mr-core-lab/ncita-protocol-checker and https://hub.docker.com/repository/docker/mrcorelab/protocol-checker/general.

**Figure 1.**
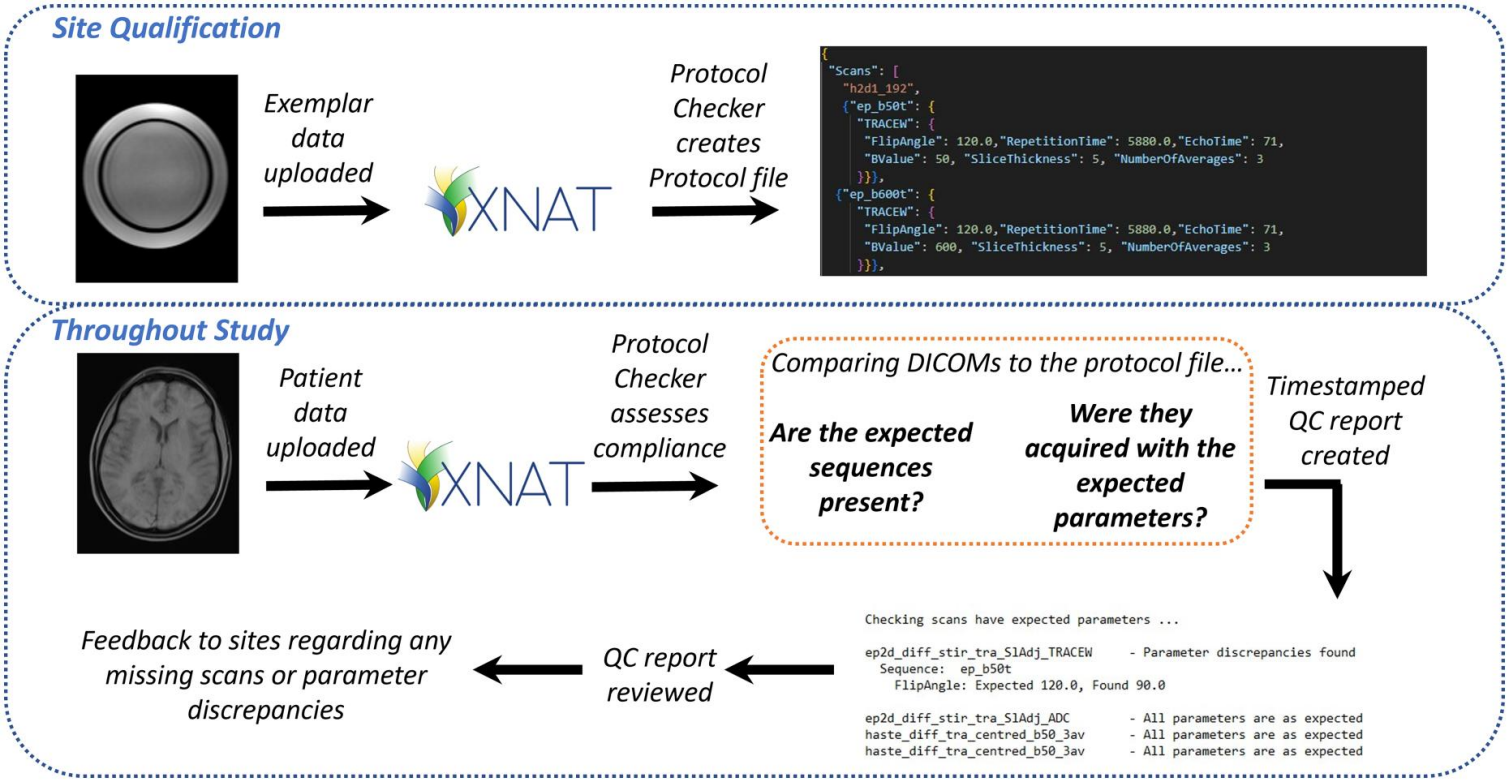
Proposed pipeline for using Protocol Checker to monitor imaging protocol compliance during a multicentre study. Exemplar phantom or volunteer data are uploaded to XNAT during the site qualification process and used, alongside the imaging protocol, to generate the site-specific master file, listing expected series and expected values of the pre-determined parameters. Protocol Checker is then launched on each subsequent dataset uploaded by that site, generating output files that summarize imaging protocol deviations. The actions of the 2 functions, “Generate master file” and “Scan protocol check,” are described in the text.

The proposed pipeline for using Protocol Checker in a multicentre study is summarized in Figure 1. Phantom and/or healthy volunteer images acquired during site qualification are used to define the expected protocol, against which all subsequently acquired data are compared. Protocol Checker can provide on-going QC throughout the lifetime of a study, allowing rapid feedback to be provided to sites in the case of imaging protocol non-compliance.

### Clinical data

The scan protocol check was assessed on 174 WB-MRI examinations acquired across 10 UK sites. The sites used scanners from 3 manufacturers and a range of models^15^ (note that 2 of the sites described by Rata *et al*^15^ were qualified for the study but did not recruit any patients), as summarized in Table 1, and had a range of WB-MRI experience prior to site enrolment.

**Table 1.** Summary of scanner field strength, manufacturer, model, and no. of patients across the 10 sites.

Site	Field strength (T)	Manufacturer	Model	Number of patients
1	1.5	Siemens	Aera/Avanto	26
2	1.5	Siemens	Aera	22
3	1.5	Siemens	Aera/Avanto	6
4	1.5	Philips	Ingenia	8
5	3	Siemens	Skyra	9
6	1.5	Siemens	Aera	27
7	1.5	Siemens	Aera	3
8	1.5	Siemens	Aera	38
9	1.5	Siemens	Aera	33
10	3	GE	Discovery MR750w	2

Note that 2 of the sites described in Rata *et al*^15^ were qualified for the study but did not recruit any patients.

Two tiers of imaging protocol checking were performed, as defined in Table 2. Tier 1 defined an imaging protocol compliant with the comprehensive MY-RADS guidelines^13^ (excluding axial T2w fast spin-echo sequences), specifying the expected sequences and a small number of important parameters (eg, slice thickness [ST], b-values). This was followed by a more stringent compliance check, using the detailed parameter set for each site, as documented in a previous publication.^15^ This demonstration was conducted using Protocol Checker integrated into XNAT, although XNAT is not a prerequisite for the use of Protocol Checker.

**Table 2.** Summary of the expected imaging protocols for both tiers of the protocol compliance checks.

Parameter	DICOM tag	Series			
		DWI	Dixon	T1w spine	T2w spine
Tier 1—MY-RADS compliance					
Slice thickness (mm)	(0018,0050)	5	5	4-5	4-5
Orientation	(0020,0037)	Axial	Axial	Sagittal	Sagittal
b-values (s mm^−2^)	(0019,100c)	50-100 500-600 800-900	–	–	–
Tier 2—Site-specific MUKnine imaging protocol (example)					
Field strength (T)	(0018,0087)	1.5			
Model	(0008,1090)	Aera			
Manufacturer	(0008,0070)	Siemens Healthcare			
b-values (s mm^−2^)	(0019,100c)	50 600 900	–	–	–
No. averages (per b-value)	(0018,0083)	3 6 6	1	–	–
TR (ms)	(0018,0080)	6240	7.6	–	–
TE (ms)	(0018,0081)	73	2.39/4.77	–	–
TI (ms)	(0018,0082)	180	–	–	–
Flip angle (°)	(0018,1314)	90	16	–	–
Receiver bandwidth (Hz/pixel)	(0018,0095)	1964	400	–	–
Reconstructed resolution (mm^2^)	(0028,0030)	1.6 × 1.6	0.8 × 0.8	–	–

DICOM tags are given for each parameter on a Siemens scanner, although some of these vary between manufacturers. Tier 1 is derived from the MY-RADS guidelines.^13^

Abbreviations: DICOM = Digital Imaging and Communications in Medicine; DWI = diffusion-weighted imaging; MY-RADS = Myeloma Response Assessment and Diagnosis System; TE = echo time; TI = inversion time; TR = repetition time; T1w = T1-weighted; T2w = T2-weighted.

A subset of the data (121 of the 174 examinations) have previously been assessed for qualitative image quality by a radiologist.^17^ This subset constituted all the examinations that were available in the study repository at the time, with the additional examinations uploaded subsequently. Each exam was rated by a single radiologist for overall exam quality, and for the quality of the DW and Dixon imaging on a 4-point Likert scale (Excellent, Good, Suboptimal, Non-diagnostic) based on the radiologist’s assessment of the clinical quality of the imaging. In this previous study, 1 examination from each site (10 examinations in total) was randomly selected and scored again by the same radiologist and by a second radiologist in order to assess repeatability and reproducibility of the scoring. Moderate to excellent agreement was reported for all series across intra- and inter-rater scoring. These scores were used to assess the correlation between imaging protocol deviations and qualitative image quality with a Mann-Whitney U-test (performed in Python with α = 0.05).

## Results

**Figure 2.**
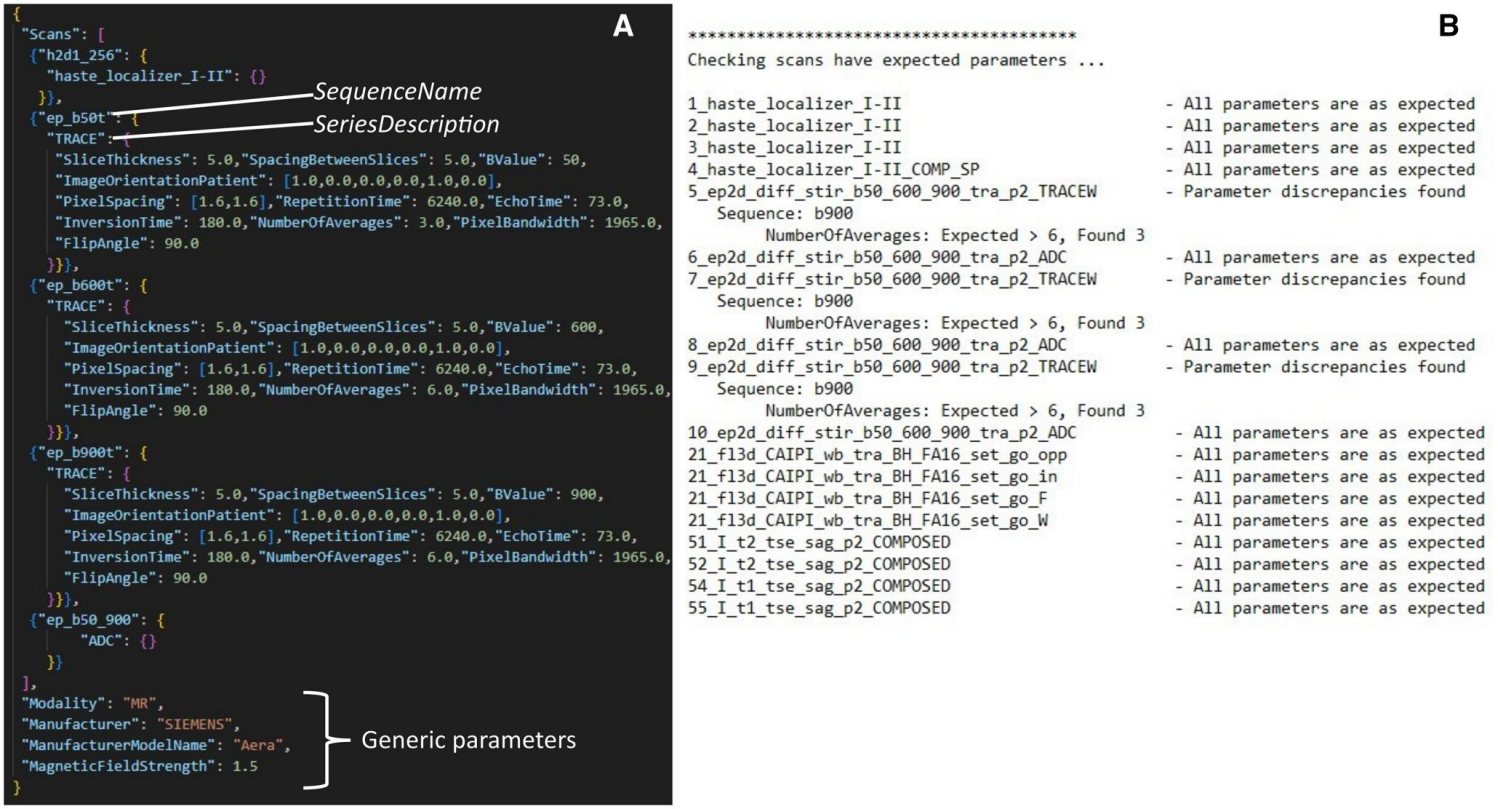
Example input and output files. (A) Extracts from the master file, defining the expected imaging protocol for a specific site. Series are identified using the *Sequence Name* and *Series Description* DICOM metadata tags and the values of each expected parameter are defined for b50, b600 and b900 DWI. The apparent diffusion coefficient (ADC) map is listed as an expected series but does not have any parameters defined. Generic parameters, which are expected to be the same for all series, are listed at the bottom of the file. (B) An extract from the results file produced by Protocol Checker, highlighting parameter discrepancies for DW and Dixon imaging.

**Figure 3.**
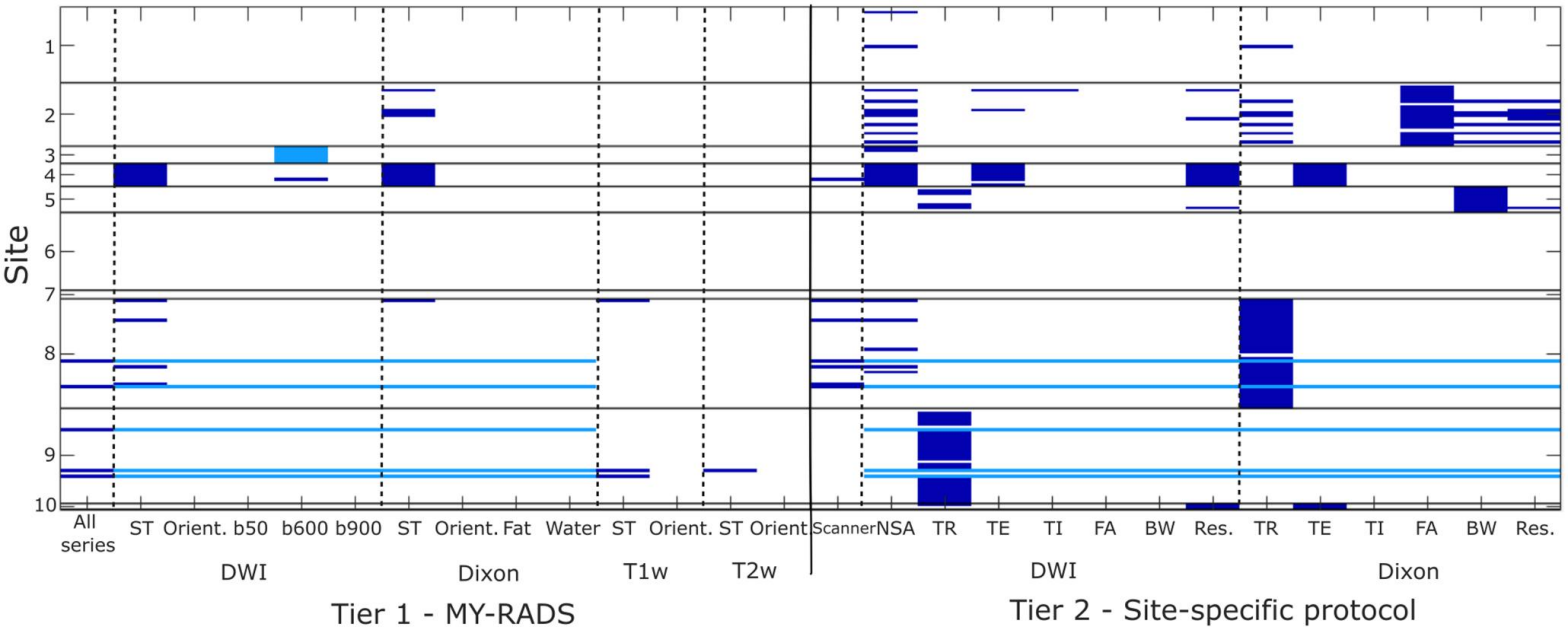
Summary of imaging protocol deviations for both tiers of compliance across all examinations at all sites. All series refers to the presence of DW, Dixon, and T1w and T2w spine imaging; Scanner refers to whether the qualified scanner for that site was used. Each column represents a checked parameter and each row represents a single examination, grouped into sites so that the height of each site’s section is proportional to the number of patients. White—the parameter matched the expected value (ie, sites 6 and 7 achieved 100% compliance), dark blue—an imaging protocol deviation was found, light blue—check was not performed (eg, some exams did not include DW or Dixon imaging, as indicated in the All Series column, so checks could not be performed for these series). Abbreviations: DWI = Diffusion-weighted imaging; MY-RADS = Myeloma Response Assessment and Diagnosis System; T1w = T1-weighted; T2w = T2-weighted.

There were no technical failures of the Protocol Checker software for any of the 174 examinations in the MUKnine dataset. Extracts from example imaging protocol and results files are shown in Figure 2. Imaging protocol deviations are summarized in Figure 3 and Table 3 for the 2 tiers of compliance checks.

**Table 3.** Summary of imaging protocol compliance.

			Number of exams with expected value	% compliant	Range of found values
Tier 1		All series present	169	97.1	–
	DWI	Slice thickness	157	92.9	5-7 mm
		Orientation	169	100.0	–
		b-value = 50 s mm^−2^	169	100.0	–
		b-value = 600 s mm^−2^	162	99.4	–
		b-value = 900 s mm^−2^	169	100.0	–
	Dixon	Slice thickness	156	92.3	5-7 mm
		Orientation	169	100.0	–
		Fat	169	100.0	–
		Water	169	100.0	–
	T1w spine	Slice thickness	171	98.3	3-5 mm
		Orientation	174	100.0	–
	T2w spine	Slice thickness	173	99.4	3-5 mm
		Orientation	174	100.0	–
	Full MY-RADS compliance		153	87.9	–
Tier 2		Correct scanner	167	96.0	–
	DWI	No. averages	144	85.2	b50: 1-4 b600: 2-6 b900: 2-7
		TR	138	81.7	7.0-14.5 s
		TE	160	94.7	70-83 ms
		TI	168	99.4	**1.5T:** 160-180 ms **3T:** 260-260 ms
		FA	169	100.0	–
		BW	169	100.0	–
		Resolution	156	92.3	1.6 mm × 1.6 mm-3.4 mm × 3.4 mm
	Dixon	TR	127	75.1	4.2-7.6 ms
		TE	159	94.1	1.8-4.9 ms
		TI	169	100.0	–
		FA	150	88.8	10-22°
		BW	154	91.1	400-1032 Hz/pixel
		Resolution	160	94.7	0.8 mm × 0.8 mm-1.3 mm × 1.3 mm
	Full specific protocol compliance		70	40.2	–

Abbreviations: BW = bandwidth; DWI = diffusion-weighted imaging; FA = flip angle; MY-RADS = Myeloma Response Assessment and Diagnosis System; TE = echo time; TI = inversion time; TR = repetition time; T1w = T1-weighted; T2w = T2-weighted.

The vast majority (96.0%) of exams were acquired using a scanner that was qualified for use in the study at that site. Complete compliance with the MY-RADS guidelines (tier 1) was found for 87.9% of examinations, with 4 sites achieving complete compliance for all exams. The most common deviations noted were incorrect ST for DWI and Dixon imaging: 92.9% and 92.3% compliance across all exams, respectively. DW and Dixon imaging were not acquired for 2.9% of exams, whilst 0.6% (a single exam) included DWI with 2 b-values, instead of 3.

Deviations from the site-specific imaging protocols were more common, although compliance was greater than 75.0% for all parameters. Complete compliance with the site-specific protocol (tier 2) was found for 40.2% of exams and 2 sites did not have any deviations for any of their exams. Common deviations included number of averages for DWI (85.2% of all exams were compliant) and repetition time (TR) for DWI and Dixon: 81.7% compliance and 75.1% compliance, respectively.

**Figure 4.**
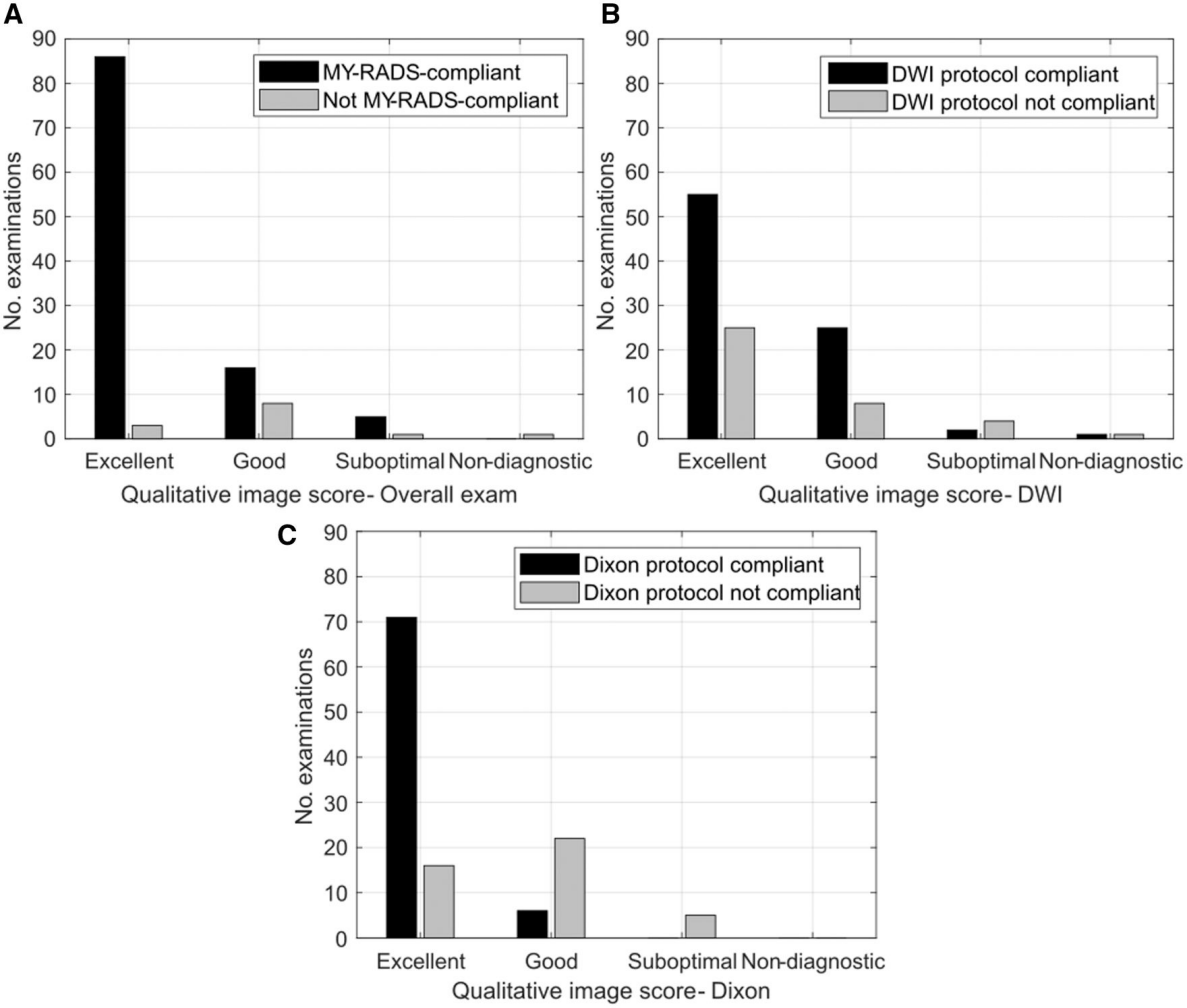
The relationship between imaging protocol compliance and qualitative radiological image scoring. An examination was regarded as compliant if it was 100% compliant with the MY-RADS guidelines (tier 1), the DWI protocol (tier 2), or the Dixon protocol (tier 2) for A, B, and C, respectively. Abbreviations: DWI = Diffusion-weighted imaging; MY-RADS = Myeloma Response Assessment and Diagnosis System.

Figure 4 illustrates the relationship between imaging protocol compliance and qualitative radiological image scoring for the subset of 121 examinations for which qualitative scoring was available. A Mann-Whitney U test showed there was a statistically significant difference in image quality score between exams with and without 100% compliance for overall exam score (U = 702, p < 0.01) and Dixon imaging score (U = 1785, p < 0.01). No statistically significant difference was found for DWI score (U = 1577, p = 0.70).

## Discussion

Standardized imaging protocols are crucial to achieve consistent image quality,^18^^,^^19^ greater reproducibility in quantitative measurements,^20^ and increased statistical power in multicentre studies.^21^^,^^22^ Protocol deviations should be avoided as they may lead to inaccurate measurement of quantitative values, poor reproducibility, and potential rejection of data. In real-world settings, it may be justified to modify imaging protocols, but these deviations should be recorded so they can be considered at the time of analysis. Persistent unplanned deviations may be indicative of issues at a site, for example, inadequate staff training or a saved protocol with incorrect parameters, which can be addressed if highlighted.

Protocol Checker is a novel software solution for automatic monitoring of imaging protocol compliance through an imaging repository, highlighting missing series and parameter deviations in an efficient and integrated way. Although demonstrated retrospectively here, this pipeline is intended to be applied prospectively in future studies to identify recurring issues with protocol compliance and enable corrective action to be taken.

While commercial protocol adherence software solutions are already in use, there are limited open-source packages available. In many studies, bespoke code is developed by the study team to monitor imaging protocol compliance; however, this code is not generalizable to other studies. One open-source solution is available to assess protocol differences across large datasets^6^; however, Protocol Checker is novel as it is easily configurable by the user, has been integrated into an imaging repository, and is designed to be used prospectively on individual examinations as they are uploaded. Integration into an imaging repository allows Protocol Checker to form part of a generalizable data QC workflow in which it can be set up to rapidly produce reports and feedback to sites to have a meaningful impact on study outcomes.

The previous finding of good overall image quality in the MUKnine dataset^17^ is complemented by the finding here that most sites were able to conduct most examinations according to the MY-RADS guidelines. The site qualification process, which established imaging protocols at all sites, was crucial to this outcome.^15^ The sites enrolled in MUKnine reflect the variation in scanner hardware and software, as well as site expertise that would be found across general clinical practice, demonstrating the versatility of the software.

As expected, good imaging protocol compliance was associated with higher image quality for overall examinations and Dixon imaging. This finding should be regarded as an indicator of the importance of protocol compliance and not as a rigorous evaluation of the causes of inferior image quality in WB-MRI, as the quality assessment included only a limited consideration of repeatability. Although not explicitly evaluated here, it is reasonable to assume that higher image quality is associated with improved lesion detection and more accurate radiological assessment. The effect of imaging protocol deviations on quantitative measurements was not evaluated in this demonstration. Previous studies have assessed this however, with findings including that apparent diffusion coefficient (ADC) measurements are highly dependent on the choice of b-values^23^ and acquisition sequence.^24^ The literature on this subject has informed the definition of acceptable parameter ranges for ADC measurement with DWI in the brain, liver, prostate and breast,^25^ which Protocol Checker could be used to monitor adherence to.

This work did not assess the effect of protocol deviations on quantitative measurements such as ADC, but these are known to be sensitive to differences in acquisition parameters.^20^ The use of Protocol Checker on these retrospective data will inform the set-up of future WB-MRI studies, which we propose will lead to better imaging protocol compliance and more consistent image quality in the future. Not all parameter deviations are equally significant for the quantitative outcomes of the study and there is scope to incorporate this into the use of Protocol Checker by grading deviations in terms of their importance. Where necessary, this could be used to provide criteria for the exclusion of exams that were not acquired with the expected imaging protocol to preserve the robustness of study outcomes.

Some of the deviations identified here appear to be isolated incidents that can be attributed to a mistake or a necessary modification for the specific patient, but other deviations occurred more consistently. For example, all the Dixon imaging from Site 4 was conducted with a ST of 7 mm (rather than 5 mm). This is indicative of a more persistent problem at that site where an incorrect imaging protocol is being repeatedly selected, perhaps due to inadequate staff training. In this case, the persistent deviations were not identified until the study was complete, but real-time use of Protocol Checker would have allowed quick, automatic identification of deviations and feedback to be provided to the site after the first patient was scanned. The mistakes could then have been rectified in time for the next patient’s scan, minimizing the number of sub-optimal examinations and improving the consistency of the data. Although the software is demonstrated here for a WB-MRI study, Protocol Checker is intended to be a versatile and extensible software solution that is not specific to any single technique or modality. As it only interacts with the DICOM header, the software is applicable to any imaging data that uses the DICOM format, including other MRI techniques and other imaging modalities such as CT or PET. A limitation of Protocol Checker is that it requires an exemplar examination acquired with the correct imaging protocol to create the master file for subsequent examinations. A useful extension of this work would be to generate the master file from the imaging protocol files on the MR scanner, allowing differences between acquired data and saved protocols to be identified. The present implementation of the software relies on matching either the Series Description or Sequence Name (or equivalent) DICOM tags of a series to those found in the master file. If the relevant tags are removed during the anonymization process, the software will not be able to match series in the new data to those in the master file and will fail. Series classification approaches have been developed that match features in the images themselves rather than relying on metadata,^26^^,^^27^ and future work could look to improve the robustness of Protocol Checker by incorporating these.

The software requires the user to understand the format of the DICOM header during the initial generation of the master file. This may be challenging as many important features of the acquisition and reconstruction are reported differently by different scanner manufacturers and between different software versions. This is a particular problem for image reconstruction parameters as these are often recorded inconsistently. Public DICOM tags are highly unlikely to change between software versions, although vendor-specific private tags are liable to change. In these cases, the code will need to be proactively developed to accommodate changes as they occur.

The workflow proposed here is dependent on data being uploaded to a repository soon after acquisition to allow issues to be addressed quickly and may also depend on standardized anonymization and data transfer procedures to ensure relevant DICOM tags are not removed. An alternative future approach would see the software implemented in-line on scanners, allowing it to run on data pre-anonymization and provide real-time feedback on imaging protocol compliance. Protocol Checker is limited to monitoring aspects of the acquisition that are recorded in the DICOM header. Other important features, such as RF coil configuration or contrast administration timings, are not recorded in the DICOM header and therefore cannot be monitored using Protocol Checker. This study assessed the correlation between protocol compliance and radiological image quality; however, the importance of good compliance was not considered in relation to quantitative measurements or lesion detection.

## Conclusions

Protocol Checker is presented as an open-source piece of software for conducting automated monitoring of compliance with an agreed imaging protocol. It is demonstrated in a multicentre study of WB-MRI in myeloma to evaluate protocol compliance, identifying which imaging parameters frequently deviated. This allows sites to be individually evaluated in terms of protocol compliance and, when used in real-time, will allow corrective action to be taken when a site persistently deviates.

This software will provide a valuable step in a standardized data QC routine, alongside other containers for automated data QC and analysis through an imaging repository.^28^ The goal of this infrastructure is to support greater standardization of image acquisition, analysis, and QC in multicentre studies to support the clinical translation of quantitative MR imaging biomarkers.
